# Three-Year Contraceptive Failure Rates During the HER Salt Lake Contraceptive Initiative

**DOI:** 10.1001/jamanetworkopen.2026.17273

**Published:** 2026-06-16

**Authors:** Jessica N. Sanders, Gentry Carter, Brooke W. Bullington, Alexandra Gero, Rebecca Simmons, Lori M. Gawron, Jennifer E. Kaiser, David K. Turok

**Affiliations:** 1 ASCENT Center for Reproductive Health, Department of Obstetrics and Gynecology, University of Utah, Salt Lake City

## Abstract

**Importance:**

Differences in typical-use contraceptive failure rates between long-acting reversible contraception (LARC; such as intrauterine devices [IUDs] and implants) and shorter-acting methods (depot medroxyprogesterone acetate [DMPA], pills, rings, and male condoms) are often the focus of contraceptive counseling, but assessments of contraceptive counseling have not focused on contraceptive effectiveness over time.

**Objective:**

To assess 3-year continuation and typical-use contraceptive failure rates for 7 reversible contraceptive methods provided with access barriers removed.

**Design, Setting, and Participants:**

The HER Salt Lake Contraceptive Initiative was a 3-year prospective longitudinal cohort study (September 2015 to March 2017, with follow-up data collected through June 2020). Participants (new contraceptive users 18-45 years who indicated they wanted to avoid pregnancy for at least 1 year) enrolled at 4 family planning clinics in Salt Lake County, Utah, and received person-centered contraception counseling and same-day access to the reversible contraceptive method of their choice. Data were analyzed from June 2024 to February 2026.

**Exposure:**

The exposure was contraceptive method selected at baseline (copper IUD, DMPA, implant hormonal IUD, condoms, pill, or ring).

**Main Outcomes and Measures:**

The outcome was experiencing a contraceptive failure, defined as an unintended pregnancy (self-reported or identified through electronic medical record) experienced while using a contraceptive method in the previous 4 weeks. Method-specific continuation and failure rates were calculated using a life table analysis.

**Results:**

Among 4275 contraceptive users (1759 [41%] aged 20-24 years), 96 pregnancies resulting from contraceptive failures of methods initiated at baseline were identified. Of all participants, 529 (11%) selected a copper IUD, 558 (13%) selected DMPA, 823 selected an implant (19%), 1025 (24%) selected a hormonal IUD, 52 (<1%) selected condoms, 1065 (25%) selected pills, and 223 (5%) selected the ring. Cumulative continuation at 3 years included 741 hormonal IUD users (72%), 455 implant users (55%), 321 copper IUD users (61%), 186 DMPA users (33%), 75 ring users (34%), 376 pill users (35%), and 8 male condom (15%). Three-year contraceptive failure rates per 100 person-years were 0.7 (95% CI, 0.4-1.1) for hormonal IUD users, 0.8 (95% CI, 0.5-1.3) for implant users, 1.1 (95% CI, 0.6-1.8) for copper IUD users, 1.1 (95% CI, 0.6-2.1) for DMPA users, 1.4 (95% CI, 0.6-3.2) for ring users, 1.6 (95% CI, 1.1-2.3) for pill users, and 2.6 (95% CI, 0.5-10.0) for male condom users.

**Conclusions and Relevance:**

In this cohort study of individuals initiating a contraceptive method following person-centered contraceptive counseling and removal of access barriers, low 3-year contraceptive failure rates were observed for all methods, and shorter-acting methods had lower failure rates than previously reported typical use rates. These findings suggest that removing access barriers to preferred contraceptive methods may support access to clinician-dependent LARC methods, like IUDs and implants, and improve the contraceptive effectiveness of user-controlled, shorter-acting methods.

## Key Points

**Question:**

With person-centered contraceptive counseling and same-day method access, what are 3-year continuation and typical-use failure rates for reversible contraceptive methods?

**Findings:**

In this cohort study of 4425 contraceptive users, 96 pregnancies resulted from baseline method failure. Three-year continuation ranged from 15% (male condom) to 72% (hormonal IUD), and 3-year pregnancy rates were low across methods, including shorter-acting methods such as injection, pill, or ring.

**Meaning:**

These findings suggest that removing access barriers may reduce typical-use failure rates for shorter-acting methods compared with historical estimates, supporting counseling that centers patient preference and method continuation.

## Teaser

This cohort study assesses 3-year continuation and typical-use contraceptive failure rates for 7 highly effective reversible (HER) contraceptive methods.

## Introduction

Most sexually active people of reproductive age use 1 or more contraceptive methods to support their human rights to control their fertility and enjoy their sexuality. Effectiveness is one of many essential factors influencing contraceptive decision-making.^1^ Contraceptive counseling includes information about perfect use and typical use. Perfect use reports efficacy, or how well a contraceptive method prevents pregnancy during a clinical trial, with strict protocol adherence, extensive inclusion and exclusion criteria, and close monitoring of correct and consistent use.^2^ Efficacy trials include participant reimbursement, adherence prompts, and limited follow-up, rarely exceeding 1 year.^3^

Typical use effectiveness, on the other hand, accounts for barriers to obtaining contraceptive methods that require clinician visits, including pharmacy refills for pills, patches, and rings; subsequent clinic encounters for injectable intramuscular depot medroxyprogesterone acetate (DMPA); or frequent purchasing of condoms. Typical use effectiveness also accounts for individual user experiences, including missing pills and imperfect use.^4^ The National Survey of Family Growth is the most widely used source of contraceptive effectiveness data. It draws on an ongoing, nationally representative sample of reproductive-age individuals residing in the US and includes information on contraceptive use at the time of last pregnancy.^5,6,7^

The Contraceptive CHOICE Project^8^ provides a rare and important example of a prospective effectiveness evaluation of multiple methods. The study examined 9256 participants for 2 to 3 years with excellent participant follow-up. CHOICE provided long-acting reversible contraception (LARC)-focused counseling and allowed participants to overcome common access barriers such as cost and method availability. Of all participants, 77% selected an intrauterine device (IUD) or contraceptive implant. Those who selected contraceptive pills, patches, or rings had a pregnancy rate of 4.55 per 100 participant-years, 20 times higher than the LARC users.^8^ CHOICE LARC methods had pregnancy rates approximating ideal use, and user-dependent methods had pregnancy rates that were multiples higher than ideal use but still lower than those reported by the National Survey of Family Growth (9%).^9^ This research generated clinical practice changes focusing on increasing LARC access and provision to decrease unintended pregnancy rates.

While evaluations of the LARC-focused approach found reductions in unintended pregnancy, it also identified infringements of autonomy and trust in the health care system, aggravating existing disparities already more common for racial and ethnic minority communities, youths, and those with resource challenges, including undocumented immigrants and incarcerated people.^10,11,12^ From these critiques grew efforts to provide patient-centered contraceptive counseling, which incorporates the individual’s unique concerns and focuses on the issues of greatest concern to the user.^13,14^ To date, outcome assessments of patient-centered contraceptive counseling focus on the quality of care and method selection, with little attention to impacts on contraceptive effectiveness over time.^15,16^

From January 2015 to December 2017, the HER Salt Lake Contraceptive Initiative addressed these gaps, building on the work conducted by CHOICE and other state-based contraceptive initiatives.^9,17,18^ HER Salt Lake provides a unique opportunity to assess contraceptive continuation and pregnancy risk over 3 years among participants who received a intervention protocol that included patient-centered counseling and same-day access to 7 different highly effective reversible (HER) contraceptive methods, including an initial 3-month supply to pills, rings, and condoms, and the ability to come back to get refills or switch for the duration of the study without out-of-pocket cost. Additionally, HER Salt Lake employed a rigorous and comprehensive assessment of pregnancy outcomes through electronic health records (EHRs) and linkage to state vital records.

## Methods

### Study Design

We designed the HER Salt Lake Contraceptive Initiative to prospectively assess contraceptive method uptake, switching and discontinuation, and pregnancy risk in a cohort of contraceptive users who received no-cost care for same-day reversible contraceptive methods, counseling focused on their priorities, and the ability to return and change methods without cost over 3 years (September 2015 to March 2020, with follow-up data collected through June 2020). The protocol is described in detail in a previous publication.^19^ This cohort study was approved by the University of Utah IRB and registered in clinicaltrials.gov (NCT02734199). Reporting follows the Strengthening the Reporting of Observational Studies in Epidemiology (STROBE) reporting guideline for cohort studies.

All participants received a standardized evidence-based, person-centered contraceptive counseling that included the Ten Best Practices Contraceptive Counseling Protocol.^20,21^ Prior to project launch, all clinical staff at participating sites were trained in this approach. Counseling included a visual representation with tiered effectiveness, as well as questions and answers about each method of interest. Participants could access their preferred method the same day and received a 3-month supply of pills and rings, their desired number of condoms, and all future medication refills, injections, or condoms at no cost for the study duration. The initiative did not cover tubal ligation, vasectomy, or contraceptive patches; participants desiring those methods were referred to outside clinics. Eligible participants included English- and Spanish-speaking individuals assigned female at birth, aged 18 to 45 years, seeking contraceptive care who had a desire to avoid pregnancy for at least 1 year. Participants all completed an oral and written consent process prior to enrollment. We enrolled 4425 participants at their initial visit who completed an enrollment survey and agreed to complete 8 follow-up surveys at 1, 3, 6, 12, 18, 24, 30, and 36 months after enrollment.

Based on prior studies and removal of cost-related barriers, we hypothesized that method selection would shift toward more clinician-dependent methods during the initiative but that observed pregnancy rates would be consistent with previously published typical-use failure rates. Using historic clinic numbers and capacity, we anticipated enrolling at least 1540 IUDs and implants and 1190 shorter-acting method users. We determined that we would be able to detect differences in first-year failure rates between sample proportions with more than 80% power for all but 1 method (condoms). Prior publications report details of the HER Salt Lake Contraceptive Initiative, including changes in method uptake during each of the intervention periods and 3-year switching and discontinuation.^19,22^

### Data Collection and Measures

We collected secure survey data through REDCap to assess contraceptive use and pregnancy experiences among individuals using the implant, copper T380A IUD, levonorgestrel 52 mg IUD (hormonal IUD), pills, ring, DMPA, nonhormonal behavioral methods (condoms, fertility awareness–based methods, and withdrawal), and oral emergency contraception. We decided a priori that methods with fewer than 20 users at baseline would be excluded from the analysis because we would not have sufficient power to detect method-specific differences in failure rates at 1 year or beyond.

Pregnancy outcome data were triangulated from multiple sources: follow-up surveys, EHR reviews of the region’s largest abortion and obstetric clinicians, and a population database that includes birth records. On self-reported surveys, participants answered the question, “Have you had a positive pregnancy test since the previous survey?” and provided the date of the positive pregnancy test, pregnancy confirmation, and any clinic-based dating information. To identify pregnancies that were underreported on surveys, we linked the full cohort to the Utah Population Database, which includes Utah birth certificates and EHRs from the state’s 2 largest health systems.^23^ Linkage was conducted using participant names, dates of birth, and addresses. We also extracted records from the Planned Parenthood Association of Utah EHR system, where patients were initially enrolled, to determine if individuals lost to follow-up had any evidence of a pregnancy event, abortion care, or miscarriage management, and whether those with pregnancies also had documentation of continued contraceptive use. For each pregnancy identified through the Utah Population Database and EHR review, we obtained the date of the last menstrual period and the date of pregnancy confirmation. One author with expertise in family planning and obstetric care (D.T.) reviewed clinical and pharmacy records to confirm that the participant’s initial contraceptive method was in use within 2 weeks of the pregnancy identification. We determined contraceptive failures through self-reported method use and pregnancy intention. Participants who indicated that they were pregnant responded to a follow-up question, “When you got pregnant, were you trying to get pregnant?” We considered those who responded no as unintended pregnancies. We considered participants who indicated that they had used the method they received at enrollment within the last 4 weeks and had an unintended pregnancy as contraceptive failures.

While all participants reported a desire to prevent pregnancy for at least 1 year at enrollment, we also asked them to rate their emotional orientation toward pregnancy at baseline by responding to the question, “How would you feel about getting pregnant in the next month?” Responses were reported using a scale of 1 (worst feeling you can imagine) to 100 (happiest you could possibly feel).

### Statistical Analysis

We used a combination of Stata version 19 (StataCorp) and R version 4.5.1 (R Project for Statistical Computing) statistical software to carry out data management and analysis.^24,25^ We conducted a comprehensive analysis of contraceptive effectiveness using up to 36 months of follow-up for each study participant to assess typical-use contraceptive effectiveness across 7 method types.

We calculated method-specific continuation and failure rates using a life table analysis, a recommended best practice for calculating contraceptive efficacy rates.^26^ In analyses, observations were censored at pregnancy, method discontinuation or switching, or study completion. Participants lost to follow-up who did not have a unique identifier in the Utah Population Database and had no relevant EHR events were also censored at their last completed survey. We calculated contraceptive continuation rates and incidence rates with 95% CIs for contraceptive failures at 1, 2, and 3 years, as well as cumulative incidence failure rates based on reported person-years among continuers for each method. Given that other typical-use failure rate calculations account for sexual activity, we assessed robustness of our findings by performing a sensitivity analysis restricting to participants who reported sexual activity in the past 4 weeks at every follow-up survey and were not lost to follow-up (eTable 1 in Supplement 1).

We used χ^2^ and Fisher exact tests to assess whether demographic variables, including method, age, race and ethnicity, relationship status, sexual identity, religion, highest level of education, insurance status, and emotional orientation to pregnancy, were associated with any contraceptive failure. We included participant self-reported race and ethnicity as a key variable given the effects of systemic inequities that contribute to disparities in health outcomes and historically higher pregnancy rates in some groups. Race and ethnicity categories included African American or Black, American Indian or Alaska Native, Asian, Hispanic or Latina, Native Hawaiian or Pacific Islander, White, and other (any self-reported race or ethnicity not otherwise specified).

To further examine associations between methods, user characteristics, and contraceptive failure, we used univariable and multivariable Cox proportional hazard models. These models allowed us to examine differential effectiveness between the 7 contraception methods across population subgroups, including age, race and ethnicity, relationship status, sexual identity, religious affiliation, highest level of education, and insurance status. In models, the copper IUD was used as the reference method, selected alphabetically as a method-neutral choice. Using unadjusted model outputs, we constructed survival curves showing the hazard of pregnancy for each contraceptive method. Finally, we compared HER Salt Lake typical use pregnancy rates with previously published rates using a 1-sample proportion test. We assessed statistical significance at α < .05. Data were analyzed from June 2024 to February 2026.

## Results

The HER Salt Lake cohort includes 4425 participants. This analysis excludes 150 participants: 35 with incomplete enrollment surveys, 78 who reported a positive pregnancy test within 30 days of their first survey date due to recent abortion care, and 37 who either selected emergency contraception only, fertility awareness–based methods, withdrawal, patch, or no method. The analytic cohort of 4275 people (97% of consented individuals) had a mean (SD) follow-up time of 29 (14) months, with 3848 (90%) completing 1 year, 3591 (84%) completing 2 years, and 3505 (82%) completing 3 years of follow-up (eFigure in Supplement 1).

Table 1 reports participant demographics, with 1759 (41%) starting the study between 20 and 24 years of age; 134 (3%) identifying as Asian, 927 (22%) identifying as Hispanic or Latina, and 2656 (63%) identifying as White; and 3067 (72%) identifying as exclusively heterosexual. Regarding feelings about pregnancy, participants responded with a median (IQR) of 10 (2-28) out of 100, indicating a strong desire to not be pregnant. Participants most frequently selected oral contraceptive pills followed by hormonal IUDs and the implant.

**Table 1.** Participant Characteristics by Method Selected at Baseline in HER Salt Lake (N = 4275)

Characteristic	Participants, No. (%)							
	Overall (N = 4275)	CuIUD (n = 529)	DMPA (n = 558)	Implant (n = 823)	LNG IUD (n = 1025)	Condoms (n = 52)	Pill (n = 1065)	Ring (n = 223)
Age, y								
18-19	835 (20)	63 (12)	150 (27)	180 (22)	179 (17)	7 (13)	225 (21)	31 (14)
20-24	1759 (41)	201 (38)	211 (38)	379 (46)	397 (39)	26 (50)	471 (44)	74 (33)
25-29	977 (23)	150 (28)	105 (19)	174 (21)	246 (24)	12 (23)	218 (20)	72 (32)
30-34	436 (10)	68 (13)	58 (10)	58 (7.0)	117 (11)	7 (13)	97 (9.1)	31 (14)
≥35	268 (6)	47 (9)	34 (6)	32 (4)	86 (8)	0	54 (5)	15 (7)
Race and ethnicity								
African American or Black	75 (2)	7 (1)	20 (4)	18 (2)	11 (1)	1 (2)	15 (1)	3 (1)
American Indian or Alaska Native	75 (2)	9 (2)	20 (4)	12 (2)	18 (2)	0	15 (1)	1 (<1)
Asian	134 (3)	20 (4)	12 (2)	22 (3)	34 (3)	2 (4)	39 (4)	5 (2)
Hispanic or Latina	927 (22)	100 (19)	140 (26)	235 (29)	170 (17)	10 (20)	238 (23)	34 (15)
Native Hawaiian or Pacific Islander	40 (1)	4 (1)	5 (1)	5 (1)	8 (1)	0	16 (2)	2 (1)
White	2656 (63)	343 (65)	304 (56)	464 (57)	711 (70)	32 (63)	648 (62)	154 (70)
Other^a^	320 (8)	41 (8)	46 (8)	58 (7)	69 (7)	6 (12)	79 (8)	21 (10)
Relationship status								
Married or committed relationship	2516 (59)	316 (60)	321 (58)	485 (59)	619 (61)	25 (48)	618 (58)	132 (59)
Single	1478 (35)	173 (33)	202 (36)	279 (34)	340 (33)	25 (48)	384 (36)	75 (34)
Other^a^	270 (6)	39 (7)	34 (6)	57 (7)	64 (6)	2 (4)	58 (6)	16 (7)
Sexual identity								
Exclusively heterosexual	3067 (72)	352 (67)	418 (75)	582 (71)	728 (71)	33 (63)	788 (74)	166 (74)
Mostly heterosexual or bisexual	1035 (24)	158 (30)	104 (19)	196 (24)	273 (27)	18 (35)	235 (22)	51 (23)
Another sexual identity	173 (4)	19 (4)	36 (7)	45 (6)	24 (2)	1 (2)	42 (4)	6 (3)
Religion								
Religious	1116 (32)	123 (27)	144 (34)	215 (32)	273 (30)	10 (26)	294 (36)	57 (31)
Not religious	2166 (62)	310 (67)	245 (58)	419 (62)	593 (65)	26 (67)	469 (58)	104 (57)
Other^a^	223 (6)	31 (7)	30 (7)	42 (6)	44 (5)	3 (8)	52 (6)	21 (12)
Prefers not to answer	770 (18)	65 (12)	139 (25)	147 (18)	115 (11)	13 (25)	250 (23)	41 (18)
Education								
Associates, vocational, technology degree	1717 (41)	225 (43)	199 (37)	315 (39)	440 (43)	20 (40)	425 (40)	93 (42)
Bachelor’s degree or higher	672 (16)	116 (22)	42 (8)	102 (13)	207 (20)	9 (18)	155 (15)	41 (19)
High school or less	1824 (43)	181 (35)	304 (56)	392 (48)	368 (36)	21 (42)	473 (45)	85 (39)
Insurance								
None or unknown	2020 (51)	215 (43)	279 (55)	369 (49)	451 (46)	19 (41)	579 (59)	108 (51)
Parents	923 (23)	120 (24)	102 (20)	203 (27)	246 (25)	12 (26)	198 (20)	42 (20)
Private	849 (21)	143 (29)	91 (18)	149 (20)	221 (23)	13 (28)	181 (18)	51 (24)
Public	184 (5)	22 (4)	32 (6)	35 (5)	52 (5)	2 (4)	30 (3)	11 (5)
Pregnancy orientation variable, median (IQR)^b^	10 (2-28)	8 (2-24)	14 (4-32)	9 (2-28)	7 (1-23)	16 (2-37)	13 (3-34)	15 (3-30)

Abbreviations: CuIUD, copper intrauterine device; DMPA, injectable depot medroxyprogesterone acetate; LNG IUD, levonorgestrel intrauterine device.

^a^ Participants’ self-reported race and ethnicity, relationship status, and religion all included an other category. Participants who responded other were prompted to self-describe their racial and ethnic identity, relationship status, and religion.

^b^ Participants report their emotional orientation toward pregnancy at baseline by responding to the question, “How would you feel about getting pregnant in the next month,” using a scale of 1 (worst feeling you can imagine) to 100 (happiest you could possibly feel).

Among the 4275 individuals, 529 (11%) selected a copper IUD, 558 (13%) selected DMPA, 823 (19%) selected an implant, 1025 (24%) selected a hormonal IUD, 52 (<1%) selected condoms, 1065 (25%) selected pills, and 223 (5%) selected the ring. Three-year cumulative contraceptive continuation of the method selected at baseline was as follows: 741 (72%) for hormonal IUDs, 321 (61%) for copper IUDs, 455 (55%) for implants, 376 (35%) for pills, 75 (34%) for rings, 186 (33%) for DMPA, and 8 (15%) for condoms. Overall, 96 method failures were identified among individuals continuing their original method. We identified 90 pregnancies using HER Salt Lake survey results and an additional 6 pregnancies through the Utah Population Database and Planned Parenthood Association of Utah EHRs.

Three-year cumulative incidence failure rates per 100 people (Table 2) were (1.7; 95% CI, 1.0-2.7) for hormonal IUDs, (1.8; 95% CI, 1.1-3.1) for implants, (2.5; 95% CI, 1.4-4.3) for the copper IUD, (2.0; 95% CI, 1.0-3.6) for DMPA, (2.7; 95% CI, 1.1-6.0) for the ring, (3.0; 95% CI, 2.1-4.3) for pills and (3.8; 95% CI, 0.7-14.0) for male condoms. This is visualized in the Figure, which presents the survival curve for unadjusted hazard of pregnancy by baseline method. eTable 2 in Supplement 1 combines methods into LARC (hormonal and copper IUDs and implants), all shorter-acting methods (injection, pill, ring, and condoms), and pill and ring only. In our study, the 1-year incident failure rate was 1.0 (95% CI, 0.70-1.5) per 100 people for all LARC users and 1.6 (95% CI, 1.1-2.3) per 100 people for short-acting method users. These were not statistically different in this supported environment with multiple months’ supplies provided at no cost. Three-year contraceptive failure rates per 100 person-years were 0.7 (95% CI, 0.4-1.1) for hormonal IUD users, 0.8 (95% CI, 0.5-1.3) for implant users, 1.1 (95% CI, 0.6-1.8) for copper IUD users, 1.1 (95% CI, 0.6-2.1) for DMPA users, 1.4 (95% CI, 0.6-3.2) for ring users, 1.6 (95% CI, 1.1-2.3) for pill users, and 2.6 (95% CI, 0.5-10.0) for male condom users.

**Table 2.** Cumulative Life Table for Incident Contraceptive Failure at Years 1, 2, and 3, in HER Salt Lake^1^

Method and follow-up time	Persons at the start of interval, No.	Person months, No.	Cumulative months, No.	Discontinue or switch methods, No.	Lost to follow-up, No.	Pregnancies, No.	Cumulative pregnancies, No.	Yearly continuation rate, No. (%)	Cumulative continuation rate, No. (%)	Conception rate per cycle, %	Survival rate per y, %	Proportion still protected, No. (%)	Cumulative life table failure rate per 100 people (95% CI)
Implant													
Year 1	823	8392	8392	85	64	8	8	730 (89)	730 (89)	<1	>99	666 (81)	1.0 (0.30-1.64)
Year 2	666	5496	13 888	189	41	4	12	473 (71)	473 (65)	<1	>99	432 (53)	1.5 (0.64-2.28)
Year 3	432	4026	17 914	79	43	3	15	350 (81)	350 (55)	<1	>99	307 (37)	1.8 (0.91-2.73)
CuIUD													
Year 1	529	5361	5361	78	19	8	8	443 (84)	443 (84)	<1	>99	424 (80)	1.5 (0.47-2.55)
Year 2	424	3798	9159	82	36	5	13	337 (80)	337 (67)	<1	>99	301 (57)	2.5 (1.14-3.78)
Year 3	301	3054	12213	35	24	0	13	266 (88)	266 (61)	0	100	242 (46)	2.5 (1.14-3.78)
LNG IUD													
Year 1	1025	10581	10581	91	76	10	10	924 (90)	924 (90)	<1	>99)	848 (83)	1.0 (0.37-1.58)
Year 2	848	8514	19095	98	53	5	15	745 (88)	745 (80)	<1	>99)	692 (68)	1.5 (0.73-2.20)
Year 3	692	6900	25995	78	74	2	17	612 (88)	612 (72)	0	100	538 (53)	1.7 (0.88-2.44)
Pills													
Year 1	1065	8006	8006	345	99	20	20	700 (66)	700 (66)	<1	>99)	601 (56)	1.9 (1.07-2.69)
Year 2	601	4026	12032	215	72	9	29	377 (63)	377 (45)	<1	>99)	305 (29)	2.7 (1.75-3.70)
Year 3	305	2598	14630	97	9	3	32	205 (67)	205 (35)	<1	>99	196 (18)	3.0 (1.98-4.03)
Ring													
Year 1	223	1841	1841	62	19	3	3	158 (71)	158 (71)	<1	>99	139 (62)	1.3 (0.00-2.86)
Year 2	139	876	2717	59	12	3	6	77 (55)	77 (43)	<1	>99	65 (29)	2.7 (0.57-4.81)
Year 3	65	492	3209	21	3	0	6	44 (68)	44 (34)	0	100	41 (18)	2.7 (0.57-4.81)
DMPA													
Year 1	558	4242	4242	171	80	9	9	378 (68)	378 (68)	<1	>99	298 (53)	1.6 (0.57-2.66)
Year 2	298	1650	5892	145	33	1	10	152 (51)	152 (42)	<1	>99	119 (21)	1.8 (0.69-2.89)
Year 3	119	1008	6900	45	0	1	11	73 (61)	73 (33)	<1	>99	73 (13)	2.0 (0.82-3.12)
Condoms													
Year 1	52	224	224	34	0	1	1	17 (33)	17 (33)	<1	>99	17 (33)	1.9 (0.00-5.64)
Year 2	17	126	350	6	3	1	2	10 (59)	10 (19.2)	1	99	7 (14)	3.8 (0.00-9.07)
Year 3	7	96	446	2	0	0	2	5 (71)	5 (15)	0	100	5 (10)	3.8 (0.00-9.07)

Abbreviations: CuIUD, copper intrauterine device; DMPA, injectable depot medroxyprogesterone acetate; LNG IUD, levonorgestrel intrauterine device.

**Figure.**
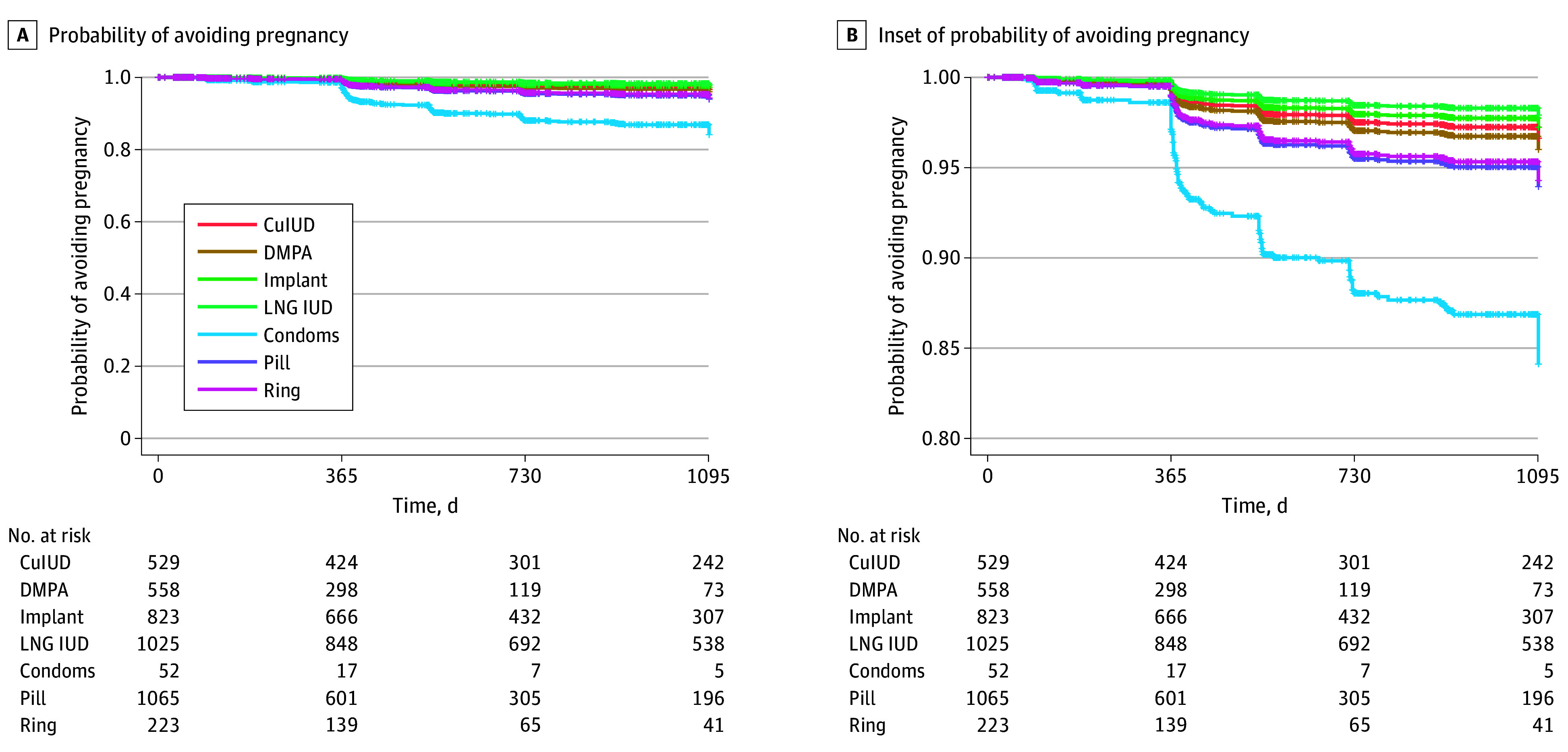
Kaplan-Meier Estimates of Pregnancy-Free Survival by Baseline Contraceptive Method Curves show the estimated probability of remaining pregnancy-free (ie, no pregnancy resulting from contraceptive failure) over follow-up time (days; 0 through approximately 1095) among participants initiating a copper intrauterine device (CuIUD), levonorgestrel IUD (LNG IUD), implant, injectable depot medroxyprogesterone acetate (DMPA), oral contraceptive pill, vaginal ring, or male condoms. Tick marks indicate censoring. The inset (B) magnifies the y-axis (0.90–1.00) to facilitate comparison between methods.

Bivariate analyses showed no difference in method distribution between those who continued their original method and experienced a contraceptive failure and those who did not (eTable 3 in Supplement 1). Furthermore, experiencing a contraceptive failure at any point during the study was not associated with race and ethnicity, relationship status, sexual identity, religion, highest level of education, or insurance status. There was a higher proportion of participants aged 18 to 19 years and a lower proportion of participants aged 35 years and older experiencing a pregnancy while using a contraceptive method, compared with those aged 20 to 24 years, but this difference was not significant. Emotional orientation toward pregnancy did not significantly differ between individuals who experienced contraceptive failures and those who did not.

In adjusted Cox models, there were no differences in pregnancy risk over time between copper IUD and injectables (hazard ratio [HR], 1.08; 95% CI, 0.41-2.84), implants (HR, 0.81; 95% CI, 0.36-1.82), hormonal IUD (HR, 0.59; 95% CI, 0.27-1.29), condoms (HR, 3.12 95% CI, 0.40-24.4), pills (HR, 1.26; 95% CI, 0.58-2.71), or the ring (HR, 1.34; 95% CI, 0.42-4.29) (eTable 4 in Supplement 1). All methods showed efficacy greater than 95% in the first year, and all methods showed efficacy greater than 90% over the 3-year study period.

Table 3 compares previously published typical-use failure estimates with the observed failure rates among HER Salt Lake participants in the first year of use. Those who used clinician-dependent LARC methods more frequently experienced pregnancy compared with previously published typical use estimates. Conversely, participants who used shorter-acting, user-dependent methods traditionally considered less effective had lower pregnancy rates than previously published typical use estimates.

**Table 3.** Comparison of Published Perfect Use, Typical-Use, and HER Salt Lake Contraceptive Failure Rates

Method	Pregnancies per 100 method users (95% CI)			*P* value^c^
	Perfect use^a^	Typical use^b^	HER Salt Lake typical use	
Implant	0.1	0.1	1.0 (0.5-2.0)^d^	<.001
Vasectomy (male sterilization)	0.1	0.2	NA	NA
IUD (hormone-releasing)	0.1-0.3^e^	0.1-0.4^e^	1.0 (0.5-1.8)^d^	.02
Tubal surgery (female sterilization)	0.5	0.5	NA	NA
IUD (copper T)	0.6	0.8	1.5 (0.7-3.1)^d^	.20
DMPA	0.2	4.0	1.6 (0.8-3.2)	.004
Combined and progestin-only pill	0.3	7.0	1.9 (1.2-2.9)	<.001
Vaginal ring	0.3	7.0	1.3 (0.3-4.2)	<.001
Male condom	2.0	13.0	1.9 (0.1-11.6)^f^	.02

Abbreviations: DMPA, injectable depot medroxyprogesterone acetate; IUD, intrauterine device; NA, not applicable.

^a^ Perfect use estimates were published in Trussell et al^28^ without 95% CIs.

^b^ Typical use estimates were published in Sundaram et al^4^ without 95% CIs.

^c^ *P* value for a test of 1 proportion in comparison with a previously published proportion, where H_0_ (HER Salt Lake typical use estimate) was equivalent to a previously published typical use rate and H_a_ (HER Salt Lake typical use estimate) was not equivalent to a previously published typical use rate for each method.

^d^ HER Salt Lake typical use estimates are higher than typical and perfect use estimates.

^e^ Ranges provided for hormonal IUD due to referece using multiple types of hormonal IUD brands.

^f^ HER Salt Lake typical use estimates are lower than both perfect and typical use.

## Discussion

This cohort study provides valuable insights into the 1-, 2-, and 3-year contraceptive effectiveness of 7 contraceptive methods, demonstrating that all methods were highly effective at preventing pregnancy in a supported clinical setting that incorporates practices of person-centered counseling; same day access; multiple months of pills, rings, and condoms; and removal of out-of-pocket cost. It builds on prior research on perfect use, typical use, and the impact of contraceptive access initiatives. HER Salt Lake had similar loss to follow-up as the CHOICE project, around 20%, and found similar 3-year contraceptive continuation rates of 72% for hormonal IUDs, 61% for copper IUDs, 55% for implants, 35% for pills, 34% for rings, 33% for DPMA, and 15% for condoms, compared with CHOICE which reported 70% for hormonal IUDs, 70% for copper IUDs, 56% for implants, 32% for pills, 30% for rings, 33% for DPMA, and did not include condoms.^8,27^ However notably, in comparing HER Salt Lake results with CHOICE, we report higher 100 participant-year pregnancy rates for LARC users (HER Salt Lake LARC, 0.6-0.9 vs CHOICE LARC, 0.2) and lower rates for pill users (HER Salt Lake, 1.0 vs CHOICE, 4.6). We observed less than a 2-fold difference between short-acting methods and LARC, compared with the previously reported 20-fold difference.^8^

Most other studies generating typical use data have primarily focused on short-term effectiveness or relied on mathematical models to estimate long-term outcomes.^28^ Our work expands the data by directly assessing pregnancy reports while using a method for up to 3 years, with high participant follow-up and the recommended life table approach.^26^ This longer-term perspective is crucial for understanding the sustained efficacy of different contraceptive methods and the factors that influence their effectiveness in everyday context. In addition, we employed highly rigorous pregnancy identification methods, including the use of vital records from the Utah Population Database and electronic medical records review; this goes a step beyond other studies.^9^

Notably, our results challenge the prevailing clinician bias toward LARCs as the most effective options.^29^ In comparing these results with the CHOICE project, we reported higher 100 participant-year pregnancy rates for LARC users (HER Salt Lake LARC, 1.0-1.5 vs CHOICE LARC, 0.2) and lower rates for pill users (HER Salt Lake, 1.9 vs CHOICE, 4.6). The differences in short-acting method pregnancy rates may, in part, be due to the greater number of pill packages provided in HER Salt Lake. The CHOICE Project prioritized LARC methods, and services for pill and short acting method users may have been less supported. Our study’s reporting of higher pregnancy rates for LARC users may be the result of multiple approaches (eg, surveys, clinical electronic medical record, and population database) to identify pregnancies that would have been lost to follow-up otherwise. Of note, we identified a higher pregnancy rate among HER Salt Lake implant users compared with CHOICE but were within the range reported in a meta-analysis of 15 efficacy studies of the method (0-1.4 per100 user years).^30^ We returned to evaluate each of these pregnancies to ensure correct assignment; no changes were made. While LARCs demonstrated high contraceptive effectiveness in our research, we found that all methods, when chosen according to patient preference and supported with same-day access and multiple months of the method, showed higher contraceptive effectiveness rates than previously reported.^4^ This finding underscores the importance of patient-centered contraceptive counseling. It supports the principle of contraceptive autonomy, which contains the key elements of informed choice, full choice, and free choice and aligns with the individual’s preferences and circumstances.^31^

Furthermore, it is essential to acknowledge that differences in contraceptive effectiveness across sociodemographic groups found in other studies may not reflect inherent biological differences but rather the impacts of systemic racism, oppression, and failures of health systems to meet diverse contraceptive needs.^32^ By providing preferred methods and support to all participants, our study may have mitigated some of these systemic barriers, resulting in more equitable outcomes.^33^

### Limitations

Several limitations of our study warrant consideration. First, attrition over the study period may have introduced bias, potentially overestimating method effectiveness if those who experienced contraceptive failure were more likely to drop out. Despite the potential pros of using multiple imputation to account for systematic missingness, the assumptions needed would have also increased bias and potential misclassification; therefore, using life tables was likely the most accurate representation of the population of people continuing a method and experiencing a failure.^26^ Additionally, we are limited by the small sample size of male condom users, especially into years 2 and 3. The Hawthorne effect—where participants modify their behavior due to awareness of being observed—may have influenced contraceptive use patterns, possibly leading to more consistent or correct use than might occur outside a research setting. For short-acting and behavior-dependent methods, we acknowledge that we measured current method use based on use in the past 4 weeks, which may not accurately reflect behavior at the time of intercourse. Additionally, our sample, which was recruited at Planned Parenthood, is not necessarily representative of all populations seeking reversible contraception or those living outside of Utah, limiting the generalizability of our findings.

## Conclusions

This cohort study found higher effectiveness rates among user-dependent, short acting methods compared with previous research in a supported environment, with protocols designed to meet user preferences and reduce barriers to continued use. As such, these findings may represent a supported typical use or best-case scenario rather than typical use while navigating real-life conditions. In conclusion, our findings provide encouraging data both for contraceptive access initiatives committed to offering a full range of options and for health policies that strengthen contraceptive coverage standards. Environments that remove barriers to preferred contraceptive methods, support access to clinician-dependent LARC methods (like IUDs and implants) and improve the effectiveness of user-controlled, shorter-acting methods should be encouraged. Future research should focus on translating these findings into scalable interventions that improve contraceptive care across health care settings.

## Notes

Supplement 1. eTable 1. Sensitivity analysis: One-year contraceptive failure rates among contraceptive continuers who report sexual activity in the last four weeks at all surveys eFigure. CONSORT participant flow diagram eTable 2. Calculation of incident and cumulative contraceptive failure rates per person-month using HER Salt Lake data at years one, two, and three, collapsing long-acting reversible contraception (LARC) methods and short-acting methods eTable 3. Bivariate associations between demographic characteristics and contraceptive failure eTable 4. Three-year multivariable Cox Proportional Hazard model for associations between demographic characteristics and contraceptive failure

Supplement 2. Data Sharing Statement
