# Supplementary material for: Three-Year Contraceptive Failure Rates During the HER Salt Lake Contraceptive Initiative
Source: JAMA Netw Open. 2026 Jun 16;9(6):e2617273. doi: 10.1001/jamanetworkopen.2026.17273 (PMC13273486; doi:10.1001/jamanetworkopen.2026.17273)
Supplement: Supplement 1. — eTable 1. Sensitivity analysis: One-year contraceptive failure rates among contraceptive continuers who report sexual activity in the last four weeks at all surveys eFigure. CONSORT participant flow diagram eTable 2. Calculation of incident and cumulative contraceptive failure rates per person-month using HER Salt Lake data at years one, two, and three, collapsing long-acting reversible contraception (LARC) methods and short-acting methods eTable 3. Bivariate associations between demographic characteristics and contraceptive failure eTable 4. Three-year multivariable Cox Proportional Hazard model for associations between demographic characteristics and contraceptive failure [file jamanetwopen-e2617273-s001.pdf]

Supplemental Online Content

Sanders JN, Carter G, Bullington BW, et al. Three-year contraceptive failure rates during the HER Salt Lake contraceptive initiative. *JAMA Netw. Open*. 2026;9(6): e2617273. doi:10.1001/jamanetworkopen.2026.17273

eTable 1. Sensitivity analysis: One-year contraceptive failure rates among contraceptive continuers who report sexual activity in the last four weeks at all surveys

eFigure. CONSORT participant flow diagram

eTable 2. Calculation of incident and cumulative contraceptive failure rates per person-month using HER Salt Lake data at years one, two, and three, collapsing long-acting reversible contraception (LARC) methods and short-acting methods

eTable 3. Bivariate associations between demographic characteristics and contraceptive failure

eTable 4. Three-year multivariable Cox Proportional Hazard model for associations between demographic characteristics and contraceptive failure

This supplemental material has been provided by the authors to give readers additional information about their work.

© 2026 Sanders JN et al. *JAMA Network Open*.

eTable 1. Sensitivity analysis: One-year contraceptive failure rates among contraceptive continuers who report sexual activity in the last four weeks at all surveys.

	Incident Unintended Pregnancy Rates (95% CI)						Cumulative Pregnancy Rates per person-year (95% CI)					
**Method**	**1-year**	**1-year among those reporting sexual activity**	**2-year**	**2-year among those reporting sexual activity**	**3-year**	**3-year among those reporting sexual activity**	**1-year**	**1-year among those reporting sexual activity**	**2-year**	**2-year among those reporting sexual activity**	**3-year**	**3-year among those reporting sexual activity**
Implant	8/823=0.01 (0.005, 0.02)	8/756=0.011 (0.005, 0.022)	4/644=0.006 (0.002, 0.017)	3/597=0.005 (0.001, 0.016)	3/403=0.007 (0.002, 0.023)	3/379=0.008 (0.002, 0.025)	8/823=0.01 (0.005, 0.02)	8/756=0.011 (0.005, 0.022)	12/1467=0.008 (0.004, 0.015)	11/1353=0.008 (0.004, 0.015)	15/1870=0.008 (0.005, 0.014)	14/1732=0.008 (0.005, 0.014)
Copper	7/529=0.013 (0.006, 0.028)	7/494=0.014 (0.006, 0.03)	4/415=0.01 (0.003, 0.026)	4/396=0.01 (0.003, 0.027)	0/287=0 (0, 0.016)	0/282=0 (0, 0.017)	7/529=0.013 (0.006, 0.028)	7/494=0.014 (0.006, 0.03)	11/944=0.012 (0.006, 0.021)	11/890=0.012 (0.007, 0.023)	11/1231=0.009 (0.005, 0.016)	11/1172=0.009 (0.005, 0.017)
LNG IUD	9/1025=0.009 (0.004, 0.017)	8/972=0.008 (0.004, 0.017)	5/826=0.006 (0.002, 0.015)	5/804=0.006 (0.002, 0.015)	2/655=0.003 (0.001, 0.012)	1/633=0.002 (0, 0.01)	9/1025=0.009 (0.004, 0.017)	8/972=0.008 (0.004, 0.017)	14/1851=0.008 (0.004, 0.013)	13/1776=0.007 (0.004, 0.013)	16/2506=0.006 (0.004, 0.011)	14/2409=0.006 (0.003, 0.01)
Pills	18/1065=0.017 (0.01, 0.027)	15/980=0.015 (0.009, 0.026)	9/588=0.015 (0.007, 0.03)	9/547=0.016 (0.008, 0.032)	3/303=0.01 (0.003, 0.031)	2/286=0.007 (0.001, 0.028)	18/1851=0.01 (0.006, 0.016)	15/980=0.015 (0.009, 0.026)	27/1617=0.017 (0.011, 0.025)	24/1612=0.015 (0.01, 0.022)	30/2154=0.014 (0.01, 0.02)	26/1898=0.014 (0.009, 0.02)
Ring	3/223=0.013 (0.003, 0.042)	3/210=0.014 (0.004, 0.045)	3/137=0.022 (0.006, 0.068)	3/129=0.023 (0.006, 0.072)	0/64=0 (0, 0.071)	0/61=0 (0, 0.074)	3/223=0.013 (0.003, 0.042)	3/210=0.014 (0.004, 0.045)	6/360=0.017 (0.007, 0.038)	6/339=0.018 (0.007, 0.04)	6/424=0.014 (0.006, 0.032)	6/400=0.015 (0.006, 0.034)
DMPA	9/558=0.016 (0.008, 0.032)	8/501=0.016 (0.007, 0.032)	0/292=0 (0, 0.016)	0/262=0 (0, 0.018)	1/119=0.008 (0, 0.053)	1/107=0.009 (0, 0.058)	9/558=0.016 (0.008, 0.032)	8/501=0.016 (0.007, 0.032)	9/850=0.011 (0.005, 0.021)	8/763=0.01 (0.005, 0.021)	10/1009=0.01 (0.005, 0.019)	9/870=0.01 (0.005, 0.02)
Condoms	1/52=0.019 (0.001, 0.116)	1/48=0.021 (0.001, 0.125)	1/17=0.059 (0.003, 0.308)	1/16=0.062 (0.003, 0.323)	0/7=0 (0, 0.439)	0/6=0 (0, 0.483)	1/52=0.019 (0.001, 0.116)	1/48=0.021 (0.001, 0.125)	2/69=0.029 (0.005, 0.11)	2/64=0.031 (0.005, 0.118)	2/76=0.026 (0.005, 0.1)	2/70=0.029 (0.005, 0.109)

© 2026 Sanders JN et al. *JAMA Network Open*.

eFigure. CONSORT participant flow diagram

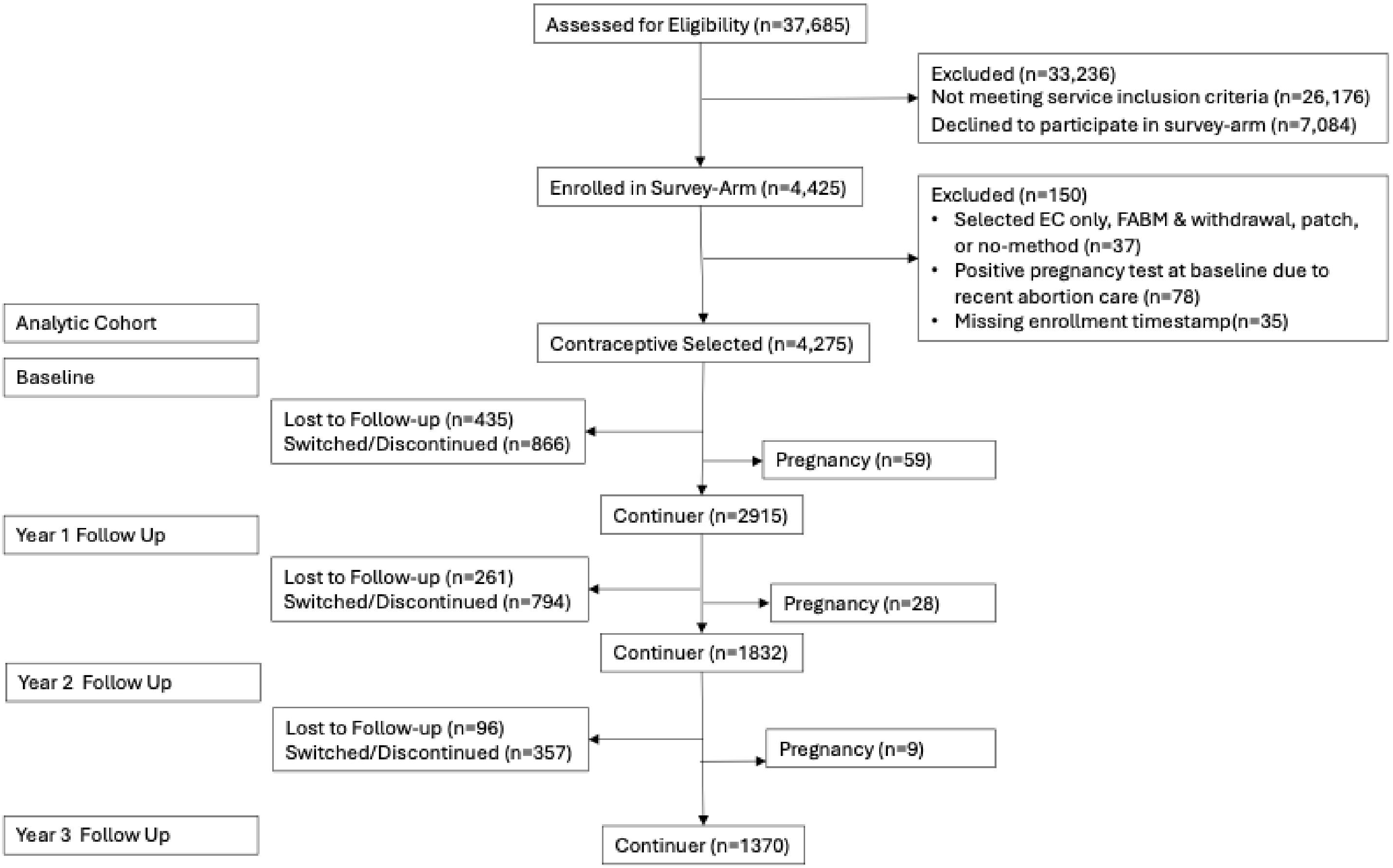

© 2026 Sanders JN et al. *JAMA Network Open*.

eTable 2. Calculation of incident and cumulative contraceptive failure rates per person-month using HER Salt Lake data at years one, two, and three, collapsing long-acting reversible contraception (LARC) methods and short-acting methods.

		LARC	All short-acting methods	p[1]	Short-acting hormonal methods (Pill + Ring only)	p[2]
Incident Unintended Pregnancy Rates (95% CI)	1-year	24/2377=0.01 (0.007, 0.015)	31/1898=0.016 (0.011, 0.023)	0.10	21/1288=0.016 (0.01, 0.025)	0.14
	2-year	13/1885=0.007 (0.004, 0.012)	13/1004=0.013 (0.007, 0.023)	0.15	12/725=0.017 (0.009, 0.03)	0.04
	3-year	5/1345=0.004 (0.001, 0.009)	4/493=0.008 (0.003, 0.022)	0.41	3/367=0.008 (0.002, 0.026)	0.50
Cumulative Pregnancy Rates per person-year (95% CI)	1-year	24/2377=0.01 (0.007, 0.015)	31/1898=0.016 (0.011, 0.023)	0.10	21/1288=0.016 (0.01, 0.025)	0.14
	2-year	37/4262=0.009 (0.006, 0.012)	44/2902=0.015 (0.011, 0.02)	0.01	33/2013=0.016 (0.011, 0.023)	0.01
	3-year	42/5607=0.007 (0.005, 0.01)	48/3395=0.014 (0.011, 0.019)	0.003	36/2380=0.015 (0.011, 0.021)	0.002

[1] Test of difference in proportions for LARC methods vs all short-acting methods

[2] Test of difference in proportions for LARC methods vs short-acting hormonal methods (pill and ring only)

© 2026 Sanders JN et al. *JAMA Network Open*.

eTable 3. Bivariate associations between demographic characteristics and contraceptive failure.

Characteristic	Contraceptive failures (n=96)	No reported failure (n=4179)	p
Method			0.32
CuIUD	13 (14%)	516 (12%)	
DMPA	11 (11%)	547 (13%)	
Implant	15 (16%)	808 (19%)	
LNG IUD	17 (18%)	1,008 (24%)	
Condoms	2 (2.1%)	50 (1.2%)	
Pill	32 (33%)	1,033 (25%)	
Ring	6 (6.3%)	217 (5.2%)	
Age			0.05
18-19	29 (30%)	1,730 (41%)	
20-24	26 (27%)	809 (19%)	
25-29	24 (25%)	953 (23%)	
30-34	14 (15%)	423 (10%)	
35+	3 (3.1%)	265 (6.3%)	
Race			0.31
African American or Black	2 (2.1%)	73 (1.8%)	
Asian	3 (3.1%)	131 (3.2%)	
Hispanic or Latina	29 (30%)	898 (22%)	
Native American or Alaska Native	1 (1.0%)	74 (1.8%)	
Native Hawaiian or Pacific Islander	1 (1.0%)	39 (0.9%)	
Race specified differently	3 (3.1%)	317 (7.7%)	
White	56 (59%)	2,600 (63%)	
Relationship status			0.26
Married/Cohabitating	62 (65%)	2,454 (59%)	
Single, dating, or other	34 (35%)	1,714 (41%)	
Sexual identity			0.44
Heterosexual	73 (79%)	2,994 (74%)	
Mostly heterosexual or bisexual	18 (19%)	1,017 (25%)	
Gay or a different sexual minority	1 (1.1%)	56 (1.4%)	
Religion			0.81
Religious	27 (35%)	1,089 (32%)	
Not religious	45 (58%)	2,121 (62%)	
Other	5 (6.5%)	218 (6.4%)	
Education			0.29
Associates, vocational/tech	34 (36%)	1,683 (41%)	
Bachelor’s or higher	12 (13%)	660 (16%)	
High school or less	48 (51%)	1,776 (43%)	

© 2026 Sanders JN et al. *JAMA Network Open*.

Insurance			0.26
None or unknown	56 (62%)	1,964 (51%)	
Parents	16 (18%)	907 (23%)	
Private	15 (17%)	834 (21%)	
Public	3 (3.3%)	181 (4.7%)	
Pregnancy orientation variable	15 (4, 31)	10 (2, 28)	0.07

© 2026 Sanders JN et al. *JAMA Network Open*.

eTable 4. Three-year multivariable Cox Proportional Hazard model for associations between demographic characteristics and contraceptive failure.

Characteristic	HR[1]	95% CI[1]	p-value
Method			
CuIUD	—	—	
DMPA	1.08	0.41, 2.84	0.88
Implant	0.81	0.36, 1.82	0.61
LNG IUD	0.59	0.27, 1.29	0.19
Condoms	3.12	0.40, 24.4	0.99
Pill	1.26	0.58, 2.71	0.56
Ring	1.34	0.42, 4.29	0.62
Age			
20-24	—	—	
18-19	1.83	0.96, 3.47	0.06
25-29	1.18	0.62, 2.27	0.62
30-34	1.28	0.56, 2.96	0.56
35+	0.49	0.11, 2.14	0.34
Race			
White	—	—	
Hispanic or Latina	1.31	0.71, 2.42	0.39
Race otherwise specified	0.96	0.46, 1.97	0.90
Relationship			
Married/Cohabitating	—	—	
Single, dating, or other	0.88	0.53, 1.44	0.60
Sexual identity			
Heterosexual	—	—	
Mostly heterosexual or bisexual	0.95	0.54, 1.68	0.87
Gay or a different sexual minority	1.19	0.16, 8.72	0.87
Religious			
Religious	—	—	
Not religious	1.07	0.62, 1.85	0.80
Other	1.29	0.48, 3.51	0.62
Education			
Associates, vocational/tech	—	—	
Bachelors or higher	0.88	0.42, 1.87	0.75
High school or less	1.02	0.59, 1.75	0.96
Insurance			
None or unknown	—	—	
Parents	0.55	0.28, 1.09	0.09
Private	0.74	0.38, 1.42	0.37
Public	0.94	0.29, 3.04	0.92

[1] HR = Hazard Ratio, CI = Confidence Interval

© 2026 Sanders JN et al. *JAMA Network Open*.
