# Supplementary material for: Three-Year Contraceptive Failure Rates During the HER Salt Lake Contraceptive Initiative
Source: JAMA Netw Open. 2026 Jun 16;9(6):e2617273. doi: 10.1001/jamanetworkopen.2026.17273 (PMC13273486; doi:10.1001/jamanetworkopen.2026.17273)
Supplement: Supplement 2. — Data Sharing Statement [file jamanetwopen-e2617273-s002.pdf]

Data Sharing Statement

Sanders. Three-Year Contraceptive Failure Rates During the HER Salt Lake Contraceptive Initiative. *JAMA Netw Open*. Published June 16, 2026.
doi:10.1001/jamanetworkopen.2026.17273

Data
Data available: Yes
Data types: Other (please specify)
Additional Information: Deidentified data can be made available upon request with IRB approval
How to access data: jessica.sanders@hsc.utah.edu
When available: With publication

Supporting Documents
Document types: None

Additional Information
Who can access the data: researchers whose proposed use of the data has been approved
Types of analyses: secondary analysis with a specific purpose
Mechanisms of data availability: with investigator support after approval of proposal and IRB
